# Use of Generics—A Critical Cost Containment Measure for All Healthcare Professionals in Europe?

**DOI:** 10.3390/ph3082470

**Published:** 2010-08-05

**Authors:** Brian Godman, William Shrank, Bjorn Wettermark, Morten Andersen, Iain Bishop, Thomas Burkhardt, Kristina Garuolienè, Marija Kalaba, Ott Laius, Roberta Joppi, Catherine Sermet, Ulrich Schwabe, Inês Teixeira, F. Cankat Tulunay, Kamila Wendykowska, Corinne Zara, Lars L. Gustafsson

**Affiliations:** 1 Institute for Pharmacological Research ‘Mario Negri’, Via Giuseppe La Masa 19, 20156 Milan, Italy; 2 Prescribing Research Group, University of Liverpool Management School, Chatham Street, Liverpool L69 7ZH, UK; 3 Department of Laboratory Medicine, Division of Clinical Pharmacology, Karolinska Institutet, Karolinska University Hospital Huddinge, SE-141 86, Stockholm, Sweden; E-Mails: Brian.Godman@ki.se (B.G.); Lars-L.Gustafsson@ki.se (L.L.G.); 4 Division of Pharmacoepidemiology and Pharmacoeconomics, Brigham and Women’s Hospital and Harvard Medical School, 1620 Tremont Street, suite 3030. Boston, MA 02120, USA; E-Mail: wshrank@partners.org (W.S.); 5 Centre for Pharmacoepidemiology, Karolinska Institute, Karolinska University Hospital, Stockholm, Sweden; E-Mails: Bjorn.Wettermark@ki.se (B.W.); Morten.Andersen@ki.se (M.A.); 6 Information Services Healthcare Information Group, NHS Scotland, 1 South Gyle Crescent, Edinburgh EH12 9EB, UK; E-Mail: iain.bishop@nhs.net (I.B.); 7 Hauptverband der Österreichischen Sozialversicherungsträger, Kundmanngasse 21, A-1031 Wien, Austria; E-Mail: Thomas.Burkhardt@hvb.sozvers.at (T.B.); 8 Faculty of Medicine, Department of Pathology, Forensic Medicine and Pharmacology, University of Vilnius, M. K. Čiurlionio g. 21/27, Vilnius, Lithuania; 9 Medicines Reimbursement Department, National Health Insurance Fund, Kalvarijų Str. 147, Vilnius, Lithuania; E-Mail: kristina.garuoliene@vlk.lt (K.G.); 10 Republic Institute for Health Insurance, Jovana Marinovica 2, 11000 Belgrade, Serbia; E-Mail: marija.kalaba@rzzo.rs (M.K.); 11 State Agency of Medicines, Nooruse 1, 50411 Tartu, Estonia; E-Mail: Ott.Laius@ravimiamet.ee (O.L.); 12 Pharmaceutical Drug Department, Azienda Sanitaria Locale of Verona, Verona, Italy; E-Mail: roberta.joppi@ulss20.verona.it (R.J.); 13 IRDES, 10, rue Vauvenargues, 75018 Paris, France; E-Mail: sermet@irdes.fr (C.S.); 14 University of Heidelberg, Institute of Pharmacology, D-69120 Heidelberg, Germany; E-Mail: ulrich.schwabe@pharma.uni-heidelberg.de (U.S.); 15 Center for Health Evaluation & Research, National Association of Pharmacies (ANF), Rua Marechal Saldanha, n.º 1, 1249-069 Lisboa, Portugal; E-Mail: Ines.Teixeira@anf.pt (I.T.); 16 Department of Pharmacology, Medical School of Ankara University, Sihhiye, Ankara 06100, Turkey; E-Mail: f.cankat.tulunay@medicine.ankara.edu.tr (F.C.T.); 17 HTA Consulting, Starowiślna Str. 17/3, 31-038 Cracow, Poland; E-Mail: k.wendykowska@hta.pl (K.W.); 18 Barcelona Health Region, Catalan Health Service, Esteve Terrades 30, 08023 Barcelona, Spain; E-Mail: czara@catsalut.cat (C.Z.)

**Keywords:** generic drugs, generic substitution, cost containment, pricing

## Abstract

Pharmaceutical expenditures in ambulatory care rose rapidly in Europe in the 1990s and early 2000s. This was typically faster than other components of healthcare spending, leading to reforms to moderate future growth. A number of these centered on generic medicines with measures to lower reimbursed prices as well as enhance their prescribing and dispensing. The principal objective of this paper is to review additional measures that some European countries can adopt to further reduce reimbursed prices for generics. Secondly, potential approaches to address concerns with generics when they arise to maximize savings. Measures to enhance the prescribing of generics will also briefly be discussed. A narrative review of the extensive number of publications and associated references from the co-authors was conducted supplemented with known internal or web-based articles. In addition, health authority and health insurance databases, principally from 2001 to 2007, were analyzed to assess the impact of the various measures on price reductions for generic omeprazole and generic simvastatin *vs.* pre-patent loss prices, as well as overall efficiency in Proton Pump Inhibitor (PPI) and statin prescribing. The various initiatives generally resulted in considerable lowering of the prices of generics as well as specifically for generic omeprazole and generic simvastatin *vs.* pre-patent loss prices. At one stage in the UK, generic simvastatin was just 2% of the originator price. These measures also led to increased efficiency for PPI and statin prescribing with reimbursed expenditure for the PPIs and statins either falling or increasing at appreciably lower rates than increases in utilization. A number of strategies have also been introduced to address patient and physician concerns with generics to maximize savings. In conclusion, whilst recent reforms have been successful, European countries must continue learning from each other to fund increased volumes and new innovative drugs as resource pressures grow. Policies regarding generics and their subsequent impact on reimbursement and utilization of single sourced products will continue to play a key role to release valuable resources. However, there must continue to be strategies to address concerns with generics when they exist.

## 1. Introduction

Pharmaceutical expenditures have increased rapidly in recent years in Europe, typically rising at between 4% and 13% per annum [1,2,3,4,5,6,7,8,9]. This is generally faster than other components of healthcare spending [6,10,11,12], similar to the US [13,14]. As a consequence, pharmaceutical expenditures in ambulatory care are now the largest or one of the largest cost components in this segment across a number of European countries [1,2,4,6,12]. In middle and lower income countries, expenditures on pharmaceuticals are also an appreciable component of expenditures, ranging from 20% to 60% of total spending on health [15].

European health authorities and health insurance organisations have instigated a number of reforms and initiatives in recent years to address this unsustainable growth. Many of the measures introduced have centred on policies surrounding generics, as they can provide high quality treatment [16] at lower costs, resulting in considerable savings [4,11,17,18,19,20,21,22,23,24,25,26]. 

The various reforms and initiatives have led to lower reimbursed prices for generics and originators as well as interchangeable brands within pharmacologic or therapeutic classes [2,4,6,19,24,25,26,27,28,29,30]. The reforms have also increased the first line prescribing and dispensing of generics where seen as standard treatment for the condition [1,4,6,11,12,17,19,27,28], the latter through, for instance, encouraging or mandating pharmacists to substitute less expensive generics in place of more expensive originators where pertinent, unless prohibited by physicians or health authorities [11,17,19,21,22,31]. Similar situations also occur in Asia. As an example, government physicians in Indonesia will soon be required to only prescribe generic drugs unless there are no generic alternatives available [32].

In 2006, generic medicines accounted for 42% of dispensed packs among 27 European countries, but only 18% of total pharmaceutical expenditures [33]. A recent analysis of 219 substances among the 27 Member States of the EU accounting for approximately 50% of prescription volumes calculated the market share of generics was approximately 30% at the end of the first year and 45% at the end of the second year [24]. Preferential co-payment policies for generics in the US among the insured population and seniors have also resulted in high utilisation of generics. As a result, generics account for approximately two thirds of prescriptions, but only 13% of costs [34,35]. 

There is appreciable variation in the utilisation of generics across Europe [36,37]. For example, there is still limited penetration of generics in Greece, accounting for only 11.6% of total pharmaceutical expenditures in 2006 [9]. There has also been appreciable differences in the reimbursed prices of generics across Europe [20,36,37], with prices of generics varying up to 36-fold across countries, depending on the molecule [20].

Pharmaceutical expenditures will continue to grow in Europe driven by demographic changes, rising patient expectations, stricter clinical targets and the continued launch of new premium priced drugs [10,24,38,39,40]. Consequently, further reforms are essential to maintain comprehensive and equitable healthcare in Europe without prohibitive increases in either taxes or health insurance premiums. 

Key areas for learning for European countries include additional measures to further lower prices of multiple source products where pertinent. They also include measures to increase the prescribing and dispensing of generics [11,12,28,29]. There have though been concerns with the effectiveness and safety of generics [4,7,9,11,21,23,25,33,34,41,42,43,44,45], with some originator companies questioning the quality of generics as part of their marketing strategies to reduce post-patent loss sales erosion [24]. However concerns with generics generally only apply to a minority of situations [9,11,34,43]. This is endorsed by two recent comprehensive reviews comparing the outcomes between generics and originators for cardiovascular diseases and epilepsy [22,46]. The authors found no evidence in published trials that originator drugs had superior effectiveness and outcomes than different generic formulations. This included drugs with a narrow therapeutic index such as propafenone and warfarin [46]. Recent studies have also shown no increase in relapse rates with generic atypical antipsychotic drugs *vs.* originators apart from initial formulations of generic clozapine in the US [47,48,49,50,51,52,53]. There has also been concerns with confusion when patients are dispensed multiple branded generics each with different names, which can potentially lead to medication errors [11]. These issues must be addressed for health authorities and insurance companies to fully capitalise on future patent losses. This is especially important with estimated global sales of USD $100B per year over the next four years subject to patent losses [54]. 

Consequently, the principal objective of this paper is to review additional measures that European countries can adopt to further reduce reimbursed prices for multiple source products where pertinent. Secondly, review potential approaches that governments, health authorities and health insurance agencies could instigate to address any concerns with generics when they arise to maximise savings. Potential measures to further enhance the prescribing of generics will be briefly mentioned and discussed further in future papers as it is recognised this is equally important to enhance prescribing efficiency.

We hope this article will stimulate debate on future measures that could be introduced as payers struggle to provide comprehensive and equitable healthcare within finite budgets. The various initiatives may also be of interest to payers outside of Europe as well as to other key stakeholder groups.

## 2. Methodology

We conducted a narrative review of articles selected from the extensive number of publications and associated references from 17 co-authors concerned with generics. These were subsequently combined with published general reviews on generics as well as additional papers and articles known to the 17 co-authors concerned with initiatives to enhance prescribing efficiency such as web-based articles that had eluded the initial selection. Finally, a targeted literature review of English language papers was subsequently undertaken by one of the authors (B.G.) across chosen European countries where the initial approaches identified no pertinent peer reviewed publications. This involved searching PubMed, MEDLINE and EMBASE between 2000 and February 2010 using key words ‘generics’, ‘generic medicines’, ‘reforms’, ‘generic reforms’, ‘generic pricing’, ‘reference pricing’ and ‘generic substitution’ and the specific country. However, no additional papers were found for possible inclusion.

The same methodological approach was adopted when collating and reviewing papers that discuss reference pricing in a class, as well as different approaches adopted by health authorities to address patient and physician concerns with generics.

There has been no review of the quality of the papers included in this paper using for instance criteria developed by the Cochrane Collaboration [55]. This is because some of the references are from non-peer reviewed journals, internal health authority documents or web based articles. Nevertheless they have been included as they were typically written by payers or their advisers, which are the principal intended audience for this paper. Table 1A in the Appendix contains the definitions used in this paper.

The generics and classes chosen for more in-depth analysis were generic omeprazole and the Proton Pump Inhibitors (PPIs)—Anatomical Therapeutic Chemical (ATC) A02BC [56], and generic simvastatin and the HMG CoA reductase inhibitors (statins)—ATC group C10AA [56]. These two classes and products were chosen as:

- They are both high volume prescribing areas in ambulatory care;
- They contain a mixture of generics, originators and single sourced products in a class;
- They are typically the subject of initiatives within countries to enhance efficiency.

The price reductions for generic omeprazole and generic simvastatin were computed by comparing reimbursed prices per Defined Daily Dose (DDDs) in 2007 or later with originator prices in 2001 or before. These dates were chosen as generic omeprazole and generic simvastatin were typically launched after 2001 among Western European countries. 

Only health authority or health insurance databases were used for the analyses in order to provide data on actual reimbursed payments for the various products in each of the two classes [36]. The sources of the administrative databases (covering all the patient population unless stated) included:

- Austria—Data Warehouse of the Federation of Austrian Social Insurance Institutions—HVB (98% of the population);
- England—Information Centre for Health and Social Care;
- Estonia—Estonian Health Insurance Fund;
- France—Medic'am database (CNAM-TS for salaried personnel covering 75% of the population);
- Germany—GAMSI-Database, the GKV Arzneimittel Schnell-Information covering all prescriptions paid by the Social Health Insurance Funds (approximately 90% of the population);
- Italy—OsMed database;
- Lithuania—Electronic database of the National Health Insurance Fund;
- Poland—National Health Fund database;
- Portugal—INFARMED (NHS) database covering approximately 75% of the population;
- Serbia—Republic of Serbia’s Health Insurance Fund database;
- Scotland—Prescribing Information System (PIS) from NHS National Services Scotland Corporate Warehouse;
- Spain—DMART (Catalan Health Service) database;
- Sweden—Apoteket AB (National Corporation of Swedish Pharmacies – monopoly up to 1 January 2010).

The concepts of ATC classification and DDDs were developed to facilitate comparisons in drug utilisation between countries [57,58]. The first comprehensive list of DDDs was first published in Norway in 1975, and has developed since then [59]. As a result, DDDs are now an internationally accepted method for comparing drug utilisation across countries especially where there are different pack sizes and possibly tablet strengths [59,60,61]. 2010 DDDs were used in line with recent recommendations [61]. 

There has been no allowance for inflation as we wanted to compute the actual impact of different policies on reimbursed prices/DDD of generics *vs.* originators over time based on the local currency. In addition, expenditure figures for the PPIs and statins are presented as percentage reductions or increases rather than actual changes in reimbursed prices or changes in overall reimbursed expenditure. This is because the extent of co-payments, wholesaler and pharmacy margins as well as taxes varies considerably across Europe. As a result, making direct expenditure comparisons difficult.

Finally, details of the reforms regarding the pricing policies for generics as well as interchangeable products in a class were taken from published sources and verified by the co-authors; alternatively, provided directly by the co-authors.

Sixteen European countries and regions have been included in this paper. These countries are: Austria, Estonia, France, Germany, Italy, Lithuania, Netherlands, Norway, Portugal, Poland, Serbia, Spain (Catalonia), Sweden, Turkey and the United Kingdom (England and Scotland). The countries were chosen to reflect differences in geography, epidemiology, financing of healthcare, available resources for healthcare, approaches to the pricing of generics, originators and single sourced products, as well as measures to enhance the prescribing of generics. 

Where possible, expenditure figures have been quoted in Euros. Current exchange rates are €1 = 1.3 US$, 1.33 CAN$, 7.84 NOK, 9.63 SEK, 0.86 GB£ (3 May 2010). 

We accept there are limitations with the study design. These include no linking of the indications and the actual doses prescribed to calculate Prescribed Daily Doses (PDD) [62], and reimbursed expenditure/PDD, as there was no access to prescribing databases. They also include the fact that no impact studies were undertaken as health authorities and health insurance agencies typically implemented a number of strategies between 2001 and 2007 to enhance prescribing efficiency making such analyses difficult to perform. In addition, most countries provided data on their total population.

## 3. Results

### 3.1. Pricing policies for generics and originals (general) and their impact

The chosen European countries have either instigated prescriptive pricing approaches for the molecule (generics and originators), let market forces drive down prices, or instigated a mixture of the two (Table 1). Table A1 (see Appendix) contains the definitions. Typically across Europe, market forces or mixed approaches appear to be the most popular methods to reduce the prices of generics [16,19,30]. Details of the different approaches are contained in Table A2, Table A3 and Table A4. 

**Table 1:** Different pricing approaches for generics and originators among exemplar European countries.

Pricing approaches	Countries
Prescriptive pricing	France, Netherlands, Norway, Turkey
Market forces	Germany, Poland, Spain*, Sweden, United Kingdom
Mixed approach	Austria, Estonia, Italy, Lithuania, Portugal, Serbia

*Spain is considering a prescriptive pricing policy for the first generic to accelerate access [63].

In Austria, the various initiatives surrounding generics and originator drugs have reduced the growth rate in ambulatory care pharmaceutical expenditure to between just under 2% to 6% per year from a baseline of 4% to 13% per year in the late 1990s and early 2000 [6]. In Catalonia, generics now account for 30% of the prescriptions by volume, helped by recent policies [12]. This is higher than a number of other regions in Spain. 

In the United Kingdom (Table A3), the introduction of the ‘Manufacturer’ and ‘Wholesaler’ scheme in 2005 to increase transparency in the cost of goods, pricing of generics and discounts given to community pharmacists, led to an average 32.4% reduction in generic prices the first full year of introduction [64,65,66]. This led to a reduction of 2% in total pharmaceutical expenditure in the England and Wales the first full year following the introduction compared with the previous year [64,65]. At one stage, the reimbursed price of generic simvastatin was just 2% of the originator price [5]. The reimbursed price of generic risperidone was also just 2% of the originator price 29 months after its availability in December 2007—10% after six months [26]. Both provide examples to other European countries on potential prices for multiple source products. In addition in 2008, savings through increased prescribing efficiency were calculated at £364M in England alone [67]. This was enhanced by high International non-proprietary name (INN) prescribing currently at over 83% of overall prescriptions, rising to over 99% for certain generics [25,27,68].

In France (Table A4), overall annual savings from the instigation of the new pricing system for generics, combined with various measures to enhance the prescribing and dispensing of generics, was calculated at €1B in 2007 [4]. This was up from €500M in 2005 [4]. In 2008, additional savings were estimated at €905mn and €1bn in 2009 [69,70]. The savings in 2007 included compulsory price cuts as well as savings from the launch of new generics, which were estimated at €340M alone in 2006 [4]. These measures helped reduce the rate of increase in ambulatory care pharmaceutical expenditure in France in recent years to between 1% to 6% per annum [4]. This was considerably lower than annual rate of increase in hospital pharmaceutical expenditure, which was approximately 20% per annum during the same period [4].

In Sweden, prices of generics fell by 40% by the end of 2005 compared with 2002 following the instigation of compulsory generic substitution. The reimbursement agency (TLV) subsequently estimated total savings from the various measures, including compulsory generic substitution, to be €700M (>SEK6.97B) from 2002 to the end of 2005 [11]. Savings are likely to be greater in recent years with reimbursed prices for high volume ambulatory care generics in 2008 at between 4% to 13% of pre-patent originator prices, *i.e.* price reductions of 87% to 96% [11]. The various combined measures led to ambulatory care expenditure on non-specialised drugs actually stabilising in Stockholm County Council in recent years [11], with the overall increase in ambulatory care pharmaceutical expenditure across Sweden limited to just 1% to 3% per annum between 2003 and 2006 [5]. This compares with an average rate of 10% per year during the 1990s and early 2000s [5]. 

A recent ecological study conducted in Stockholm (Sweden) showed no significant differences in surrogate outcomes for hypertension, diabetes or hypercholesterolaemia whether physicians adhered to guidelines including generic simvastatin, metformin or glibenclamide or chose to ignore the recommendations and prescribe drugs such as single sourced atorvastatin [71]. However there was an appreciable difference in expenditure [71].

### 3.3. Impact of pricing policies on reimbursed prices of generic omeprazole and generic simvastatin

**Figure 1:**
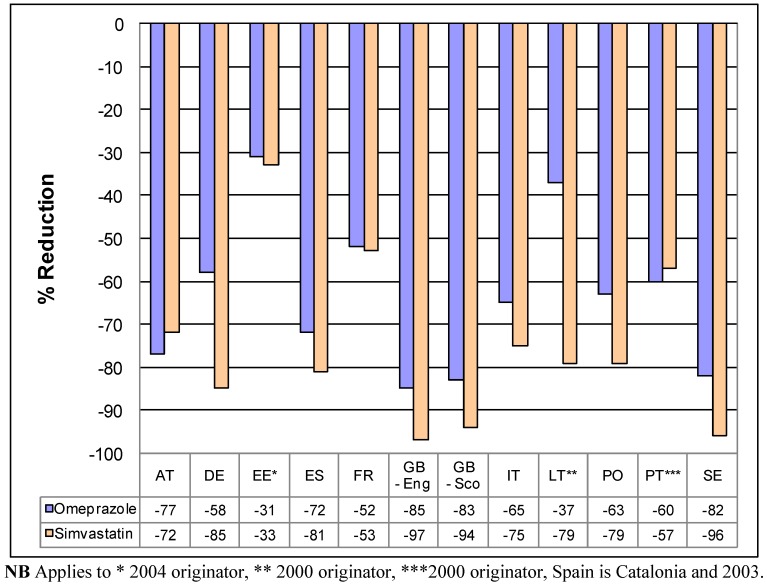
Percentage reduction in reimbursed expenditure for generic omeprazole and generic simvastatin in 2007 *vs.* 2001originator prices (unless stated) in exemplar countries.

Figure 1 depicts the impact of the different pricing approaches on reimbursed prices/DDD of generic omeprazole and generic simvastatin in 2007 compared with originator prices principally in 2001 in selected European countries. Not every country contained in Table 1 was able to provide data; however the majority were. 

There were price reductions of 16% to 20% in Serbia when reimbursed prices/DDD for generic omeprazole and generic simvastatin respectively in 2009 compared with 2004 prices. It was impossible though to compare generic prices in 2009 with originator prices in 2004 as neither originator was reimbursed in 2004. 

The patents for omeprazole and simvastatin in Italy expired in 2007. Already though in 2008, expenditure for simvastatin fell by 31% despite utilisation increasing by 17% and expenditure for omeprazole fell by 24% despite utilisation increasing by 22% [72]. 

Table 2 contains details of the overall impact of generic policies on utilisation and reimbursed expenditure in these two target disease areas. 

Studies undertaken in the UK have demonstrated that patients can be successfully switched from atorvastatin to generic simvastatin without compromising care [73,74] whilst saving an estimated £2B over five years [75]. Substantial savings were also demonstrated in the Netherlands with active switching from atorvastatin to generic simvastatin [76]. 

**Table 2:** Impact of the various measures on the utilisation and expenditure of PPIs and statins in exemplar countries in 2007 *vs.* 2001 unless stated.

Country	Change in utilisation 2007 *vs.* 2001	Change in expenditure 2007 *vs.* 2001	Additional comments
Austria—PPIs	3.6 fold increase	2.1 fold increase	Helped by voluntary price reductions for single sourced PPIs
Austria—statins	Approximately 2.4 fold increase	3% decrease	Helped by prescribing restrictions for both atorvastatin and rosuvastatin
England—PPIs	2.3 fold increase	38% reduction	Helped by the introduction of the new pricing system as well a variety of measures to enhance the prescribing of generic omeprazole *vs.* other PPIs
England—statins	5.1 fold increase	20% increase	Helped by the introduction of the new pricing system as well a variety of measures to enhance the prescribing of low cost statins *vs.* single source statins
France—PPIs	2.1 fold increase	39% increase	Helped by initiatives to enhance the prescribing and dispensing of generics *vs.* originators
France—statins	72% increase	19% increase	Helped by initiatives to enhance the prescribing and dispensing of generics *vs.* originators
Germany—PPIs	3.2 fold increase	1.4 fold increase	Helped by the introduction of reference pricing for PPIs in 2003
Germany—statins	2.1 fold increase	54% reduction	Helped by the introduction of reference pricing for statins in 2003 and the removal of atorvastatin from the normal reimbursed list
Lithuania—PPIs	32.2 fold increase	14.7 fold increase	2007 *vs.* 2000
Lithuania—statins	6.1 fold increase	1.9 fold increase	2007 *vs.* 2000
Poland—PPIs	Near doubling of the rate of increase in utilisation *vs.* expenditure		Helped by reference pricing for the PPIs
Poland—statins	4.5 fold difference in the rate of increase in utilisation *vs.* expenditure		Helped by reference pricing for the statins
Portugal—PPIs	3.8 fold increase	2.3 fold increase	2007 *vs.* 2000
Portugal—statins	5.3 fold increase	2.9 fold increase	2007 *vs.* 2000
Scotland—PPIs	2.3 fold increase	52% reduction	As England
Scotland—statins	4.9 fold increase	16% increase	As England
Spain (Catalonia) —PPIs	1.9 fold increase	7.6% decrease	2007 *vs.* 2003
Spain (Catalonia)—statins	86% increase	4% decrease	2007 *vs.* 2003
Sweden—PPIs	53% increase	49% reduction	2007 *vs.* 2000
Sweden—statins	3.2 fold increase	39% reduction	2007 *vs.* 2000

### 3.3. Reference pricing in a class

In addition to ongoing measures regarding generics and originators, just under half of the selected European countries have instigated reference pricing for products in a class (Table 3) especially where limited demand side measures to enhance the prescribing of generics [77]. This is different from reference pricing for originators and generics such as originator and generic omeprazole, *i.e.* reference pricing based on the molecule, as this applies to a class based either on pharmacological activity (ATC Level 4), such as all PPIs, all statins or all Angiotensin Converting Enzyme Inhibitors (ACEIs), or all products within a therapeutic category (ATC Level 3). Examples of the latter include all atypical antispychotics to treat schizophrenia [53] or all drugs to treat hypertension. There is also voluntary reference pricing in Austria with the potential for prescribing restrictions if manufacturers are reluctant to lower their prices once generics are available in the class [6]. Details of the various schemes are included in Table A5. 

**Table 3:** Reference pricing in classes in exemplar countries.

Country	Reference pricing in a class (pharmacologic or therapeutic)	Voluntary reference pricing
Austria		√
Germany	√	
Italy	√	
Poland	√	
Serbia	Selected products in a class	
Sweden	PPIs only—still being debated in the courts. Restrictions and delistings in recent therapeutic area reviews as more complex disease areas	
Turkey	√	
UK	Proposed by the Office of Fair Trading but rejected by the Department of Health	

This does not include external reference pricing especially for new products. Most European countries reference a number of other European countries when appraising potential reimbursed prices for new products. Prices are typically revised down if conditions change in the reference countries. This also does not include compulsory price cuts or any price: volume arrangements [78], which are in addition.

Reference pricing has also been introduced in other countries and regions outside of Europe. In 1997, reference pricing for ACEIs was introduced in British Columbia in Canada for patients aged 65 or older [13,14]. Patients have to cover the additional costs themselves for a more expensive product, which is similar to European countries. The reference price group contained three ACEIs, namely captopril, quinapril and ramipril. The other available ACEIs, benazepril, cilazapril, enalapril, fosinopril, and lisinopril, were subject to an additional co-payment of between CAN$2 to CAN$62 per month [13,14]. 

Evaluation of the scheme demonstrated that outcomes were not compromised in patients who were switched ACEIs. In addition, healthcare utilisation and associated costs outside of drug costs did not change following the reform, and patients did not discontinue their treatment as a result of the reform [13]. Drug cost savings were estimated at CAN$5.8 million the first year of introduction, some 6% of all cardiovascular drug expenditure among senior citizens in British Columbia [14]. 

### 3.4. Strategies to address concerns with generics when these occur

Health authorities and health insurance agencies have instigated a number of initiatives to address patient and physician concerns with the effectiveness and safety of generics when prescribing and dispensing them, including substitution, where these occur (Table 4) [1,2,3,4,6,7,11,25,27,43,79]. There have also been strategies in some European countries to reduce potential patient confusion when prescribed multiple branded generics. 

**Table 4:** Health Authority and Health Insurance approaches to address patient and physician concerns with generics including potential duplication.

Key Stakeholder Groups	Activities
Physicians	Option to indicate no substitution on the prescription (generally rare in practice) Only licensing generics where there are no concerns with their bioequivalence or therapeutic equivalence Encouraging INN (International non-proprietary name) prescribing from the outset even when only single sourced products are available (country and product dependent) Encouraging physicians to speak with patients where there is the potential for substitution to help allay any fears Involved with developing and adhering to an agreed list of non-substitutable products
Pharmacists	Encouraging pharmacists to speak with patients when substituting to reduce concerns (country dependent) Limiting the number of times products can be substituted where concerns Databases in pharmacies giving access to prior prescribing history to avoid potential duplication Adhering to an agreed list of non-substitutable drugs
Patients	Information and other campaigns encouraging patients to accept INN prescribing from the outset (country dependent) Promotional campaigns to allay fears regarding the effectiveness and safety of generics backed up by campaigns by health authorities and health insurance companies to enhance the acceptance of generics

Products currently excluded for substitution in Sweden include a number of anti-epileptic drugs, ciclosporin and warfarin. In Spain, non-substitutable products include carbamazapine, ciclosporin, digoxin, phenytoin, and vigabatrin. In the UK, the British National Formulary (BNF) as well as the National Prescribing Centre, have suggested that several drugs should only be prescribed by their brand name rather than by INN to enhance subsequent care as bioequivalence cannot be assumed. In addition, care with certain other products and preparations [25,64,65,80] is required including lithium, various opiods and carbamazepine.

## 4. Discussion

We believe a number of conclusions can be drawn from these findings, as well as provide guidance for the future. These include the fact that the various pricing policies for generics (Table A2, Table A3 and Table A4) have resulted in appreciable decreases in the prices of generic omeprazole and simvastatin *vs.* originator prices pre patent loss or 2000/2001 (Figure 1) in the selected European countries. As a result, releasing considerable resources to help fund increased utilisation of PPIs and statins. Sometimes, this has been at reduced overall expenditure (Table 2). Alongside this, there have also been more general savings from the availability of generics, which can be considerable, e.g. France, Sweden and the UK [4,11,27,64,67]. These savings appear to be achieved without compromising care [22,46,52,53,71]. As a result, endorsing the instigation of the various supply and demand side initiatives surrounding generics as a necessary cost containment tool to address growing budgetary pressures. 

Care though is needed in a minority of situations for health authorities and health insurance agencies to fully realise the resource benefits from the availability of generics. This includes for instance limiting or discouraging substitution for different formulations of lithium, ciclosporin, and opiods as well as certain products for the management of epilepsy. It also includes instigating prescribing databases in pharmacies, or other alternative measures, to reduce the possibility of duplication when patients are dispensed different branded generics each with different names.

We acknowledge that we have not discussed biosimilars. This is in view of the appreciable difference in effectiveness and safety data requirements for registration between oral generic small molecules and biosimilars, as well as the need for post marketing pharmacovigilance studies with biosimilars. This topic will though be discussed in future articles as biosimilars are becoming increasingly important with the biopharmaceutical market expected to grow by some 12 to 15% per year over the next few years [33,81].

As stated, further reforms are essential to ensure continued and comprehensive healthcare in Europe. Consequently, pharmaceutical companies need to appreciate and plan for significant price decreases once drugs lose their patent. This will increasingly become a pre-requisite to fund new premium priced innovative drugs. Otherwise, future patient care and commercial goals will be compromised. Likewise, physicians also need to fully appreciate the rationale behind ongoing reforms surrounding the availability of generics and work with them to fund increased volumes and new drugs within available resources.

Alongside this, European and other countries need to learn from each other. This is already happening for health reforms in general [82]. Future initiatives in some European countries could include measures to further lower prices of multiple sourced products where pertinent as well as accelerate reimbursement of generics with more frequent reviews of reimbursed prices. Austria and Norway provide examples of aggressive prescriptive pricing policies that can be introduced especially when taking into consideration their population sizes (Table A2, Table A3 and Table A4). Sweden, the UK, and more recently Lithuania, provide examples of additional measures that could be introduced to increase transparency in the pricing of generics linked with either high INN prescribing (Lithuania and the UK) or compulsory substitution unless concerns (Sweden). High INN prescribing is in line with recommendations from the WHO and International Society of Drug Bulletins [83]. Transparency with the pricing of generics is becoming increasingly important giving the extent of rebates and discounts that have, or still exist, to enhance the dispensing of particular generics [4,16,27,84]. As a result, demonstrate to health authorities the potential to further lower prices mindful though of the need to maintain a viable and sustainable market for generic manufacturers in Europe.

Compulsory generic substitution or INN prescribing is though not currently permitted in all European countries, and initiatives to increase the transparency for pricing generics is also not in operation across Europe. Possible approaches in these countries could include measures to lower or negate patient co-payment if particular branded generics are priced at a fixed percentage below the current reference price. This measure has been successfully applied in Germany. However, the potential impact will depend on the extent of the current co-payment per pack, and could be viewed as an alternative to more aggressive prescriptive pricing policies for generics. 

Other measures to conserve valuable resources alongside pricing initiatives include policies to further enhance the prescribing of generics first line through for instance economic incentives, prescribing targets and/ or prescribing restrictions for single sourced products [4,11,12,27,75,85,86]. These measures will have a direct impact in further lowering prices where market forces are used to reduce prices post patent loss. These issues will be explored in future papers.

## 5. Conclusions

The availability of generics and their increasing utilisation, combined with strategies to lower their prices, has led to considerable savings across Europe. These savings enable European health authorities and health insurance companies to provide comprehensive and equitable healthcare within finite resources. Changing demographics and the continued launch of new premium price products mandate that European countries must continue to learn from each other to further enhance efficiency given the current wide variation in reimbursed prices and the wide variation in the utilisation of generics. Care though is needed in a minority of situations when prescribing or dispensing generics to ensure savings are maximised. This may mean prescribing the originator drug in a limited number of situations. 

Different strategies are possible across Europe to enable European countries to further enhance efficiency in ambulatory care prescribing. However, possible additional measures will be country specific given the complexities surrounding prescribing in each country and the different circumstances

## Appendix

**Table A1:** Definitions used in the paper.

Term	Definition
Generics [1,2,22,25,46,87]	Generic medicines are defined as products with no intellectual property or other protection after the protection expires on the originator medicine. They have the same qualitative and quantitative composition in active substances, same pharmaceutical form, and same bio-availability as the originator medicine. Consequently, a similar therapeutic effect can be assumed There is a similar definition in the US with the FDA defining generics as bioequivalent when sufficient evidence suggests that the 90% confidence intervals for the ratio of brand-to-generic AUC (Area Under the Curve) and the maximum serum concentration fall within an acceptance ratio of 0.80-1.25 This can include branded generics since in some European countries no INN prescribing is allowed and the names of products including manufacturers have to be supplied. However, for the purposes of this paper branded generics will be incorporated in the term ‘generics’
Originators	These are the brand products pre-patent loss as well as the continuing brand name after patent loss
Pricing approaches for generics	***Prescriptive pricing for generics****-* this can be either for the molecule (generic and originator) or just for the generic. Health Authorities or Health Insurance Companies mandate price reductions that are necessary for the first generic or generics to be reimbursed. This is either based on originator prices pre-patent loss, reimbursed prices of generics in other European countries or a mixture of the two ***Market forces for generics*** - In some European countries there is no established price reduction level for the first generic or generics to be reimbursed. This is left to market forces with a number of measures in place to accelerate price reductions ***Mixture of prescriptive pricing and market forces*** - In some European countries price reductions are mandated for the first generic or generics. Market forces after that to further drive down prices

**Table A2:** Prescriptive pricing approaches for generics in selected European countries.

Country	Pricing initiatives
France [4]	Reforms were introduced in 2006 whereby generics had to be at least 50% cheaper than the originator product to be reimbursed, with prices of current off-patent medicines reduced by a further 15% to 19% for continued reimbursement Recent reforms have reduced this to 55% below originator prices for reimbursement; this reduces by 7% after 18 months Alongside this, a flat rate reference price [Tarif forfaitaire de responsabilité - TFR] was established if multiple sources exist for the same molecule but the market share of the generics was not sufficient (initially established at less than 45% penetration) with patients having to cover any additional costs themselves for a more expensive product than the generic/ reference priced product with INN prescribing increasingly encouraged
Netherlands [19,88]	The prices of generics are based on the average price of originators and generics (same active substance, strength and dosage form) in Belgium, France, Germany and the UK Pharmacists are actively encouraged to substitute by being able to retain 33% of the price difference between the current reference price for the molecule and the prescribed product
Norway [43,89]	In January 2005, the Norwegian authorities instigated a step wise approach to the pricing of generics as well as introducing incentives for generic substitution in pharmacies Under this scheme, an automatic 30% price reduction was expected for the first generic *vs.* the maximum price of the originator pre-patent loss This increases to 55% or 75% below pre-patent loss prices 6 months following the availability of the first generic depending whether annual reimbursed sales were less than or greater than 100M NOK respectively for the previous year A third step is initiated 12 months or more after the second step with discounts increasing from 55% to 65% of the originator price for products with reimbursed sales between 15 and 30M NOK in the previous year, and 80% for sales between 30M and 100M NOK There is a maximum 85% discount for products with reimbursed sales great than 100M NOK in the previous year The step model has been altered twice since its introduction with the latest change implemented in January 2008 The stepped price is the maximum price paid by the National Insurance System. Only if the prescriber forbids substitution will the Insurance Scheme refund the higher price for the originator product (if this exists). In addition, pharmacists are obliged to inform patients of the cheapest available product
Turkey	The first generic must be priced no higher than 66% of the originator’s pre-patent loss price to be reimbursed (setting the reimbursed price for the molecule) Subsequently, subject to a 11% discount the following year to continue to be reimbursed (similar for the originator)

**Table A3:** Market Forces approaches to the pricing of generics in selected European countries.

Country	Pricing initiatives
Germany [26,90]	The prices of the three most expensive products for the molecule are averaged out, divided by 3, and added to the average price of the three cheapest products to set the reference price As a result in April 2008,the reference price for ZOCOR (originator simvastatin) was €84.13 for 100 x 20 mg (down from €193.12 pre-patent loss) and generic simvastatin €62.45 Patient co-payment for the pack is abolished if the reimbursed price of the dispensed product is at least 30% below the current reference price to further drive down prices
Poland [2]	The first generic must be priced lower than the current reference price for the originator product to be reimbursed. Subsequent generics will not be reimbursed if the requested price is higher than the current cheapest generic price in the therapeutic group Market forces subsequently drive down prices of generics and originators with patients paying an additional co-pay for a more expensive branded generic or originator on top of the standard co-pay for the pack Price cuts are in addition. For instance a universal price cut of 13% was implemented in July 2006 Manufacturers and wholesalers may subsequently offer discounts or free goods to community pharmacists to have their products differentially dispensed especially if this helps reduce patient co-payments Physicians may forbid substitution if concerns
Spain [12,63,91]	There is currently no prescriptive pricing policy for generics in Spain. However, generics and originators are included within a single reference class (homogeneous group) once generics are available Reimbursed prices for the molecule are based on the average price of the three cheapest products in the group (homogeneous groups). This can be generics and originator products or just generics Since 2003, there is no opportunity for patients to pay for a more expensive product than the reference price, and since 2007 substitution with the cheapest product is mandatory (typically a generic). The cheapest product now establishes the reference price for the molecule with further price cuts envisaged as a result of recent reforms There are also ongoing discussions to accelerate reimbursement of the first generic if the requested price is at least 30% below the originator price pre-patent loss
Sweden [5,11]	Currently there is no formal prescriptive pricing policy for generics in Sweden. However, since 2002 there has been mandatory generic substitution apart from a minority of exceptions Patients pay the price difference if they wish a more expensive product apart from the product areas/ classes where there is currently no substitution (decided by the Medicines Product Agency)
UK [24,25,26,27,29,64,65,66,68,84]	There is free pricing of generics in the UK. However, there are high rates of INN prescribing in the UK averaging 83% across all products rising to over 99.5% once generics are available, e.g. generic simvastatin Under the new ‘M’ (Manufacturer) and ‘W’ (Wholesaler) scheme for the pricing and reimbursement of generics, manufacturers have to regularly report their production costs with wholesalers and pharmacists regularly reporting current discounts and rebates to enhance transparency. Prior to this, generic manufacturers offered discounts up to 80% or greater to community pharmacists to preferentially stock and dispense their generic There is currently no regulation in the UK for manufacturers to reduce originator prices once generics are launched. The OFT proposal for a maximum 25% above the generic price for reimbursement was rejected in favour of universal price cuts and the introduction of value based pricing (also refer to Table A5)

**Table A4:** Mixed approaches to the pricing of generics in exemplar European countries.

Country	Pricing initiatives
Austria [6,86,92]	The first branded generic must be a minimum of 48% below the originator product, the second branded generic 15% lower than the first, and the third branded generic 10% lower than the second. This subsequently establishes the reference price for all branded generics and the originator product Subsequent generics must be priced lower than the previous generic for reimbursement, e.g. at least 10cents/ pack The price of the originator must be 60% below the price pre-patent loss within 3 months of entry of the third branded generic to continue to be reimbursed. There are no regulations for subsequent reductions as more branded generics are launched
Estonia	The first generic must be at least 30% below the originator price to be reimbursed; the second generic 10% below this price and the next two generics 5% below the previous reference price to be reimbursed All subsequent generics must be priced lower than the last generic or the reference price to be reimbursed Originator prices must be below the generic price if companies wish to add the originator product to the reimbursement list once generics are available
Italy [93,94]	The first generic has to be at least 20% below the originator price to be reimbursed. This sets the reimbursed price with patients obliged to cover any additional cost themselves Subsequent generics must be priced lower than the current reference price to be reimbursed, with the lowest price product establishing the new reference price Patients are required to cover the additional cost for a more expensive product than the reference price with substitution by pharmacist allowed since 2001
Lithuania	Previously, the first generic must be at least 30% below originator prices to be reimbursed. This subsequently established the reimbursement rate for the molecule (originator or generic) with patients having to cover any additional costs for a more expensive product unless exempt from substitution Market forces after that to drive down prices as more generic versions of the molecule are launched Since 1 July 2004, all reimbursed prescriptions should be written by INN name with pharmacists obliged to stock the cheapest product and inform the patient of any co-pay differences to further drive down generic prices. Physicians though can still prescribe the brand name where concerns The regulations tightened in 2010, with originator name prescribing only allowed for biological drugs unless permission is granted from the State Patient Fund. In addition from1st May 2010, all pharmacists are obliged to provide data on prices to patients via computer screens Also from 2010, the second and third generics launched must be at least 10% cheaper than the first generic to be reimbursed if market forces have not driven down the prices of successive generics to this level. In addition where more than three products with the same INN are reimbursed, the originator must not be priced higher than 60% above the cheapest generic for continued reimbursement. This will drop to a maximum of 30% in 2011
Portugal [1,3]	The first generic must be priced at least 35% below the originator medicine (with the same strength and pharmaceutical form). This reduces to 20% if the originator price is below €10/ pack Prices were further reduced in 2005 and 2007 (as part of general price reductions). Price reductions were set at variable rate to stimulate competition between generics and originators depending on the market share of each active substance (applied only once and cumulative): ○ 5% for generics with a market share between 50% and 60% ○ 4% with a market share between 60% and 70% ○ 3% for generics with a market share exceeding 70% A further price reduction of 30% was introduced in October 2008 for those generics that were approved prior to April 2008 Every new generic entering the homogenous group (same active substance, dosage, pharmaceutical form and package containing at least one generic and originator) must be priced at least 3% lower than current cheapest generic product which has a market share of at least 10% in the homogeneous group Reference prices for the molecules are reviewed 4 times/ year and correspond to the highest unitary retail price of all available generics in each homogeneous group (Reference price system for the molecule - RPS - was introduced in 2002 with the first list published in March 2003). Patients must pay the difference for a more expensive brand if this exists compared with the most expensive generic Since 2002, physicians are obliged to prescribe by INN name once multiple sources exist. Pharmacists are also able to substitute where physicians have prescribed by INN and the physician has not prohibited substitution, and should inform patients about generic prices *vs.* originators. However currently no incentives or sanctions to encourage physicians to prescribe generics, and no financial incentives by law encouraging pharmacists to actively substitute
Serbia	The first generic must be priced at least a minimum of 80% of average current prices in three reference countries (Slovenia, Croatia and Italy) Subsequent generics should be priced similar or lower to gain market share with the lowest price product establishing the reference price for the molecule Originator and generic drugs must now have the same price to be reimbursed, i.e. no opportunity for patients to pay an additional co-payment for a more expensive product

**Table A5:** Countries with reference pricing for the class among the selected European countries and the impact where known.

Country	Reference pricing initiatives and impact
Austria [6,86]	Voluntary reference pricing was instigated in Austria in 2002 Under this scheme, the HVB (Federation of Austrian Social Insurance Institutions) seeks voluntary price reductions from Companies who have single sourced products in a class once generics are available. The alterative is delisting (100% co-payment) or prescribing restrictions (typically with prior authorisation schemes) Accumulated savings were €209.2M in 2006 excluding VAT
Germany [28,29]	Formula use for reference pricing in a class (e.g. PPIs and statins): There is a complicated formula for calculating the reference price for single source and branded generic products in Level 2 reference groups (drugs grouped by comparable pharmacological and therapeutic activities - ‘Jumbo classes’) in Germany This is set at the top of the lowest third of products in a class with patients required to pay any difference above the reference price Table 2 documents the overall impact of this approach for the PPIs and statins
Italy [29,95]	The reference price for the class (ATC classification – fourth level) is set at the level where the accumulated number of DDDs consumed for the class is 60% of the total market and the accumulated NHS expenditure is 50% of the total market. The only exception is where a single active substance accounts for 50% of the total market. In this case, the reference price is calculated at 15% above the cheapest active substance Products are delisted if companies do not wish to lower prices to the reference price New products in a class are exempt from reference pricing if they demonstrate significant health gain compared with current standards
Poland [2,53]	Reference pricing for interchangeable medicines in a class has been in existence in Poland since 1998 Reference groups are based on ATC levels 3 to 5, i.e. medicines having the same active substance, same pharmacological class or same therapeutic class The reference price is set at the level of the cheapest medicine in the group based on the price per dosing unit with patients funding any additional costs themselves for a more expensive product Reference groups can be large, i.e. different atypical antipsychotics
Serbia	There is currently therapeutic reference pricing for certain products within classes in Serbia, e.g. selected PPIs Patients have to pay the additional costs themselves on top of any existing co-payment for the package
Sweden [5,11,29,96]	The Swedish Reimbursement agency is currently reviewing the value of nearly 2000 medicines contained within 49 classes (by ATC classification) that are currently included within the reimbursement system. The objective is to enhance the efficient use of resources especially as more standard drugs become available as generics The reimbursement agency concluded that all PPIs have the same therapeutic effect with the exception of esomeprazole which at higher doses appears to give a better outcome in the treatment of heartburn with oesophageal reflux However since a range of PPIs were needed, the authorities granted a premium of up to 25% above generic omeprazole for continued reimbursement realising an estimated 175mn SEK/ year Several manufacturers subsequently complained about this initiative and the courts are still reviewing the situation Other disease areas are seen as more complex. As a result, reference pricing has been superseded by delistings and prescribing restrictions where concerns with the value of certain products
Turkey	The maximum reimbursement ceiling for a product in a class once reimbursed is subsequently established is 15 % above the cheapest drug in the same therapeutic equivalent group
UK (25,27,29,67,97,98)	The Office of Fair Trading in the UK proposed a 50% premium for single sourced products in a class to conserve resources building particularly on the proposed initiative for PPIs in Sweden. This was estimated to save £574.7M annually for selected interchangeable products However, the proposal was rejected in favour of price cuts as well as value based pricing for new products. In addition, further programmes among GPs to enhance the prescribing of low cost alternatives first line including the ‘Better Care, Better Value Indicators’ Alongside this, the Department of Health is also looking at introducing generic substitution as part of the Pharmaceutical Price Regulation Scheme negotiations
